# Kaumātua Mana Motuhake Pōi: a study protocol for enhancing wellbeing, social connectedness and cultural identity for Māori elders

**DOI:** 10.1186/s12877-020-01740-3

**Published:** 2020-10-02

**Authors:** Brendan Hokowhitu, John G. Oetzel, Mary Louisa Simpson, Sophie Nock, Rangimahora Reddy, Pare Meha, Kirsten Johnston, Anne-Marie Jackson, Bevan Erueti, Poia Rewi, Isaac Warbrick, Michael P. Cameron, Yingsha Zhang, Stacey Ruru

**Affiliations:** 1 University of Waikato, Private Bag 3105, Hamilton, 3240 New Zealand; 2 Rauawaawa Kaumātua Charitable Trust, 50 Colombo St., Hamilton, 3204 New Zealand; 3 University of Otago, PO Box 56, Dunedin, 9054 New Zealand; 4 Massey University, Private Bag 11 222, Palmerston North, 4442 New Zealand; 5 Auckland University of Technology, Private Bag 92006, Auckland, 1142 New Zealand

**Keywords:** Kaupapa kaumātua, Tuakana-teina, Matauranga Māori, Positive ageing, Cultural dissonance, Community-based participatory research, Mana motuhake, Hauora

## Abstract

**Background:**

The Aotearoa New Zealand population is ageing accompanied by health and social challenges including significant inequities that exist between Māori and non-Māori around poor ageing and health. Although historically kaumātua (elder Māori) faced a dominant society that failed to realise their full potential as they age, Māori culture has remained steadfast in upholding elders as cultural/community anchors. Yet, many of today’s kaumātua have experienced ‘cultural dissonance’ as the result of a hegemonic dominant culture subjugating an Indigenous culture, leading to generations of Indigenous peoples compelled or forced to dissociate with their culture. The present research project, Kaumātua Mana Motuhake Pōī (KMMP) comprises two interrelated projects that foreground dimensions of wellbeing within a holistic Te Ao Māori (Māori epistemology) view of wellbeing. Project 1 involves a tuakana-teina/peer educator model approach focused on increasing service access and utilisation to support kaumātua with the greatest health and social needs. Project 2 focuses on physical activity and cultural knowledge exchange (including te reo Māori--Māori language) through intergenerational models of learning.

**Methods:**

Both projects have a consistent research design and common set of methods that coalesce around the emphasis on kaupapa kaumatua; research projects led by kaumātua and kaumātua providers that advance better life outcomes for kaumātua and their communities. The research design for each project is a mixed-methods, pre-test and two post-test, staggered design with 2–3 providers receiving the approach first and then 2–3 receiving it on a delayed basis. A pre-test (baseline) of all participants will be completed. The approach will then be implemented with the first providers. There will then be a follow-up data collection for all participants (post-test 1). The second providers will then implement the approach, which will be followed by a final data collection for all participants (post-test 2).

**Discussion:**

Two specific outcomes are anticipated from this research; firstly, it is hoped that the research methodology provides a framework for how government agencies, researchers and relevant sector stakeholders can work with Māori communities. Secondly, the two individual projects will each produce a tangible approach that, it is anticipated, will be cost effective in enhancing kaumātua hauora and mana motuhake.

**Trial registration:**

Australia New Zealand Clinical Trial Registry (ACTRN12620000316909). *Registered* 6 March 2020.

## Background

*“A hallmark of wellbeing for older Maori is the capacity to provide leadership and direction, despite advancing years, and regardless of socio-economic position” (Sir Mason Durie, p. 1142)* [1]

The Aotearoa New Zealand (hereafter, ‘Aotearoa’) population is ageing and numerous studies demonstrate that with this phenomenon comes health and social challenges including chronic conditions, cancer, end-of-life issues, social isolation and limited opportunities for intergenerational connections [2]. More relevant to this article are the significant inequities that exist between Māori and non-Māori around poor ageing and health [3–5]. These inequities are due to structural discrimination such as unjust and unequal distribution of social determinants (e.g., income, education, housing) and a colonial history that resulted in cultural dissonance due to coercive and assimilatory policies that led to loss of language, culture, epistemologies and land [6, 7].

For scholars of indigeneity, the effects of colonisation on the wellbeing of Indigenous cultures, communities and individuals are well known, researched and documented and are, unsurprisingly, consistent across colonial contexts [8–17]. As a recent article bringing together practitioners and health scholars from multiple colonial contexts summarizes: “Globally, health disparities between Indigenous and non-Indigenous populations are ubiquitous and pervasive, and are recognized as being unfair, avoidable, and remediable (p. 512)” [18]. Similarly, the negative impact of colonisation on Indigenous life-course is internationally endemic. Typically, Indigenous peoples die considerably earlier than their non-Indigenous compatriots, creating a great sense of loss and source of pain for cultures that view their elders as bearers of knowledge critical to survivance [19]. As articulated by well-known Australian Aboriginal activist and academic, Mick Dodson: “The statistics of shortened life-expectancy are our mothers and fathers, uncles, aunties and elders who live diminished lives and die before their gifts of knowledge and experience are passed on. We die silently under these statistics (p. 11)” [20].

Although historically kaumātua (Māori elders) have faced a dominant society that has failed to realise their full potential as they age, Māori culture has remained steadfast in upholding elders as, “carriers of culture, anchors for families, models for lifestyle, bridges to the future, guardians of heritage, and role models for younger generations (p. 14)” [21]. The present research programme, Kaumātua Mana Motuhake Pōī (KMMP), is part of the Ageing Well National Science Challenge in Aotearoa (https://www.ageingwellchallenge.co.nz/), which looks to provide more focus on positive ageing as part of the government’s strategic approach to science investment. KMMP builds upon the significant innovations in Māori and Indigenous health knowledge [22–35], including research from the recently completed Kaumātua Mana Motuhake (KMM) project [36–38]. Whilst it is clear that significant disparities exist between Māori and non-Māori around poor ageing and health outcomes [3–5, 39], which in turn implicate individual, economic, social and cultural costs [39–43], this research identifies a knowledge gap in relation to this disparity and Māori culture’s veneration of elders.

### Kaumātua Mana Motuhake and cultural dissonance

Mana motuhake is a concept that foregrounds independence and autonomy to achieve actualisation—including collective determination and independence. In this manner, kaumātua assert their independence and autonomy so they can live a life of longevity and quality for self and others [15]. The current programme is invested in upholding tino rangatiratanga (independence and autonomy) and mana (status and prestige as viewed by self and others) and, accordingly, it values older people in all settings and views their experience and status as key tools for positive ageing. Furthermore, this research is grounded in Māori epistemologies surrounding ageing [44] and provides insights into how Māori epistemologies and practices surrounding ageing have the potential to improve life-courses in Aotearoa generally.

Whilst the research is grounded in a strengths-based approach, it does not assume that the kaumātua tikanga (cultural practices of elders) is consistent, practiced or even understood at a basic level by all kaumātua. Indeed, although the majority of health research on Indigenous peoples simply fails to acknowledge the negative causative effects of colonization [18], the present research programme recognizes that the majority of kaumātua of this particular generation have experienced cultural dissonance as a direct result of colonial policies. Many of today’s kaumātua, for example, were punished for speaking te reo Māori (the Māori language) through the colonial education system including in Native Schools [45]. Moreover, during the time that this generation of kaumātua were going through State education, Māori children were generally defined as ‘retarded’ based on Western models of developmental psychology [46, 47] with the blame being squarely located on ‘traditional’ Māori culture [48, 49]. That is, State policy was hegemonic in that it purposefully discouraged Māori children from practicing and valuing their Indigenous language and culture, whilst actively promoting the dominant non-Indigenous culture as superior [48].

In relation to the present research, the central point is that many of today’s kaumātua have experienced the history related above, including what has come to be referred to as ‘cultural dissonance’. It is the result of a hegemonic dominant culture subjugating an Indigenous culture, leading to generations of Indigenous peoples compelled if not forced to dissociate with their Indigenous culture. Indeed, there is a growing literature that not only foregrounds the effects of colonisation in relation to Indigenous health disparities, it also, in particular, assumes a causality between what is now increasingly referred to as colonial ‘historical trauma’ and epistemological violence [14, 50–68]. Put simply, it is increasingly accepted that there is a correlation between poor Indigenous health and cultural dissonance as a by-product of colonisation.

Relevant here is a unique study [1, 69]; Māori researcher Sir Mason Durie and colleagues carried out a health and wellbeing survey of 400 Māori kaumātua over the age of 60 years, finding that, wellbeing for older Māori was conceptualized:

… as an interaction between personal health perspectives and participation in certain key elements of Maori society e.g. land, language, marae … a proxy measure for ‘Maoriness’ has enabled correlations to be made between spirituality, cultural affinity, material wellbeing, general health status, and disability. In the study of older Maori, those participants who scored lowest on the cultural index scale were likely to have the worst health … In other words, a Maori view of wellbeing is closely linked to an ability to fulfill a cultural role (p. 1142) [1].

The author’s research supports the concept that cultural dissonance is a significant factor in relation to kaumātua wellbeing.

It also raises the question whether research directly engaging tikanga, te reo Māori and/or mātauranga (knowledge) will have meaningful health benefits for kaumātua [58]. Whilst not directly working with the elderly, pioneering research in Australia, the US and Canada has tested the hypothesis that Indigenous ‘cultural continuity’ and language revival can counter the losses rendered by colonisation [30, 51, 70–73]. Richard Oster, a Canadian researcher, and his team, found a positive relationship between preservation of culture and protection from diabetes for First Nations people [51]. Oster et al. made the cautious conclusion that ‘cultural continuity’ in part determined the health of Indigenous peoples.

Similarly, in the Aotearoa context, Rolleston [74] joined a growing body of recent literature relating to the significance of Indigenous language reclamation and revival [75–78]. She found that her participants learnt te reo Māori as an avenue to enhance their wellness for three reasons: (1) searching for identity, (2) searching for understanding of Māori epistemologies and, (3) the strengthening of family, children, and grandchildren. Another study demonstrates that whakawhanaungatanga (social connecting) and marae-based programmes influenced Māori participation rates and programme effectiveness for Māori in health rehabilitation [79]. Other research conducted with kaumātua in relation to ‘cultural continuity’ and health demonstrated that kaumātua actively participate in cultural practices, tribal, kin and marae roles and responsibilities, and passing on mātauranga [40, 80–82]. Such participation contributed to positive ageing, wellbeing, and engagement even when kaumātua experienced long-term or multiple health problems [83]. In sum, the limited research in this space tends to demonstrate that ‘cultural continuity’ of kaumātua impacts on health outcomes [84]. Although health research in this space is in its infancy, the broader thesis to be tested is that Indigenous cultural revitalisation will increase the wellbeing of Indigenous communities. The present research will directly investigate this concept by examining the association between kaumātua culture and health; in particular in relation to learning te reo Māori; mātauranga; and tuakana/teina (peer support underpinned by kinship).

### Research aims and objectives

The broader objective of this research is to empirically demonstrate that Indigenous cultural revitalisation will increase the wellbeing of Indigenous communities. KMMP is comprised of two interrelated projects that foreground dimensions of wellbeing within a holistic Te Ao Māori (Māori epistemology) view of wellbeing. This view incorporates dynamics of individual perspectives, participation in Māori community, and interconnectedness among spiritual, cultural, whānau (extended family), community, and material wellbeing. Both projects will focus on identified aspects of cultural continuity including te reo, tikanga, mātauranga (cultural knowledge), Māori values, cultural and whānau roles of kaumātua, and intergenerational knowledge exchange. Project 1 involves a tuakana-teina/peer educator model approach focused on increasing service access and utilisation to support kaumātua with the greatest health and social needs through. Project 2’s approach focuses on physical activity and mātauranga exchange (including te reo Māori; Māori language) through intergenerational models of learning. In addition, the research programme involves a network of 11 Māori service providers.

## Methods and design

### Kaupapa Kaumātua

Both projects in this research programme have a consistent research design and common set of methods that coalesce around the emphasis on kaupapa kaumātua. That is, research projects led by kaumātua and kaumātua providers that advance better life outcomes for kaumātua, their communities and their whānau. This research builds off an established relationship between university researchers and Rauawaawa Kaumātua Charitable Trust (‘Rauawaawa’), a kaumatua service provider of wrap around care. In additional the project employes kaupapa Māori ([85]; Smith GH: The development of Kaupapa Māori: theory and praxis, unpublished) and participatory research methods [86, 87]. Kaupapa Māori is a philosophy of research emphasising Māori worldviews, understandings and approaches and includes strong participatory elements. KMMP will be dependent upon the advice and direction given by kaumātua themselves, not least through a Kaumātua Board Advisory Group and an Expert Advisory Group, and kaumātua leadership within other kaumātua providers (see below). Hence the recourse to ‘kaupapa kaumātua’ (‘for-kaumātua-by-kaumātua’) as opposed to simply ‘kaupapa Māori’, signifying the engagement with the wealth of knowledge that already exists within kaumātua communities and the determination to provide kaumātua with access to decision-making power, oversight, guidance and input in relation to research methods, procedures, data-collection processes, and analyses. The present research also recognises the capacity of kaumātua as holders of Indigenous knowledge and directly includes research for sharing this knowledge with younger generations. Given the holistic approach, it is necessary for the research to emphasise intergenerational relationships and to also consider how cultural dissonance can impact life experiences across generations.

### Collaborative research foundation and co-design

The research foundation operationalises the principles of this research programme’s methodology. Core to this foundation are He Pikinga Waiora, the research network, advisory groups and internal research training.

#### He Pikinga Waiora and design logic

**Fig. 1:**
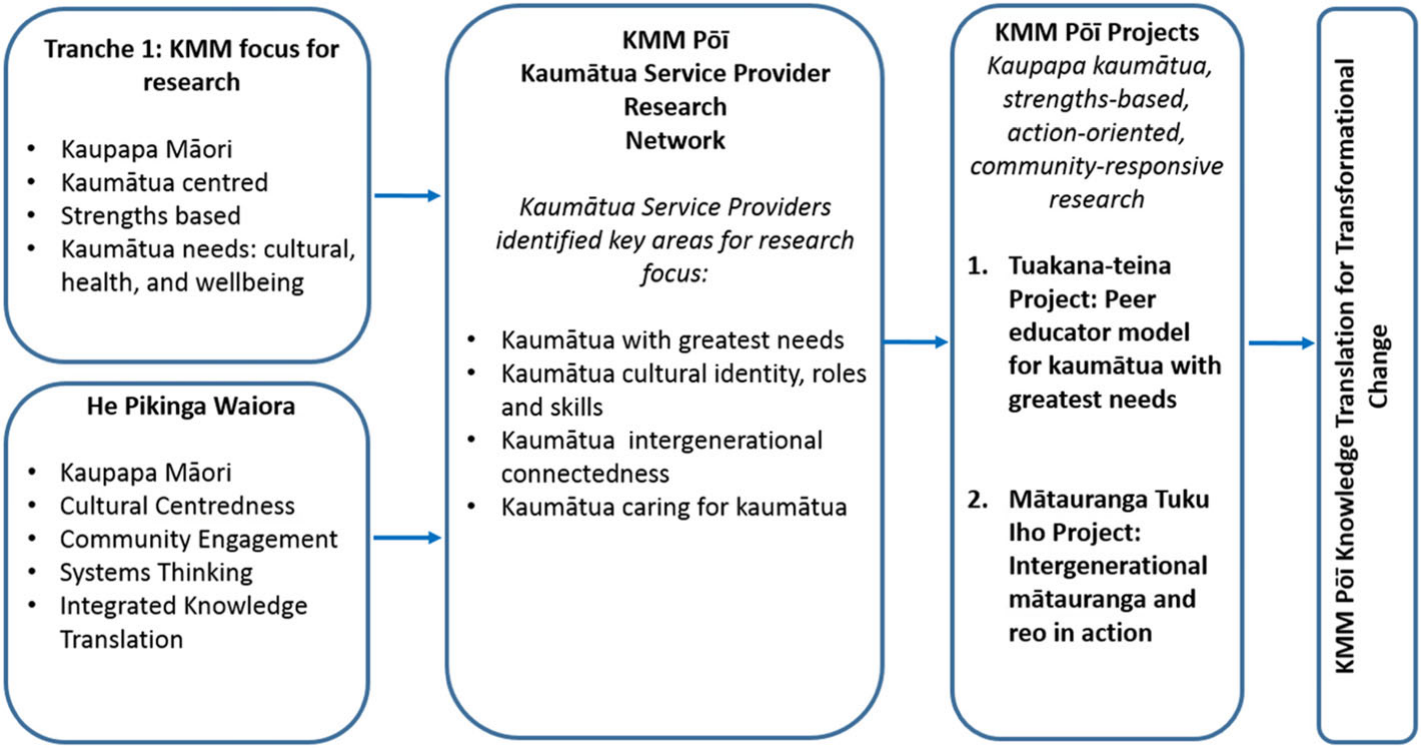
Kaumātua Mana Motuhake Pōī Research Design Logic

Figure 1 outlines the research design logic of KMMP. This research programme is guided by the He Pikinga Waiora (HPW) Implementation Framework [88]. HPW centres kaupapa Māori along with best practice from the international literature: community engagement, culture-centredness, systems thinking and integrated knowledge translation [89–91]. The framework emphasises self-determination and mātauranga Māori along with a participatory research approach that co-designs projects with end-users (both those who implement it and those who use or benefit from it). In this manner, the method facilitates the translation and uptake of research into systemic practice, thereby increasing the potential of a sustainable approach determined by kaumātua. This overall methodology reflects kaupapa kaumātua in that it ensures the programme is kaumātua and end-user led (i.e., kaumātua service providers and other stakeholders).

#### Kotahitanga research network (KRN)

A central feature of KMMP will be a research network (Kotahitanga Research Network; KRN) comprised of kaumātua service providers, researchers and other stakeholders that will facilitate collaborative identification of key kaumātua-centric research issues, help to develop the research capacity of providers, share research findings, conduct research, and generate evidence to inform policy and service development that, in turn, will impact positively on kaumātua health and wellbeing. The KRN is expected to significantly increase adoption of the research findings, given that stakeholders and partners contribute substantially to the creation of the research programme. In this respect, the KRN has the potential to magnify the impact of each project.

Research networks are formed for multiple reasons and generally consist of a group of service providers interested in using the latest evidence-based practices to improve health outcomes and/or to test innovative research approaches [92, 93]. The KRN includes 11 service providers who are affiliated with the Hei Manaaki Ngā Kaumātua National Collective of Kaumātua Service Providers and have agreed to participate in the design and testing of one of the two projects. The providers are geographically diverse, being located across the country. Meetings and consultations with providers were held prior to submission of the funding proposal to discuss the parameters of the projects and the resources needed for their participation. Input from kaumātua, providers, and researchers led to the selection of two research projects. Meetings with providers will continue throughout the projects, with 2–3 online meetings per year as well as an annual kanohi-ki-te-kanohi (face-to-face) meeting to share developments and findings.

#### Advisory groups

The use of advisory groups is consistent with HPW and kaupapa Māori principles [85, 89]. In the present research, a Board Advisory Group (BAG) for the overall research programme has already been established and Expert Advisory Groups (EAGs) for each of the two projects will also be created. The BAG comprises trustees of Rauawaawa; it has already provided and will continue to provide programme governance, oversight, guidance and input into methods, procedures, data-collection processes, and analysis. This group has served in this capacity on prior projects and applies the guiding principles upon which Rauawaawa operates as a ‘for-kaumātua-by-kaumātua’ organisation. EAGs for each project will comprise approximately six experts, including those with cultural and contextual expertise from specific providers, and experts in health and social issues of particular relevance to each project. The EAGs will meet primarily with the respective research teams, but the EAGs and the BAG will also be brought together during the first and final years to solidify the research goals and ensure wider dissemination.

#### Training for researchers

Senior members of the research team, in collaboration with the BAG, will conduct a training workshop for all researchers (including service provider/community researchers) involved in the research programme. The training aims to create consistency and collective understanding within the team regarding the research and will address the skills necessary to establish and support kaumātua participation and data collection. Specifically, training will focus on: a) Kaupapa Māori research and HPW principles; b) Research ethics and the principles of Whānau Ora; c) Needs of kaumātua within the research environment (e.g., working with kaumātua with impaired hearing and physical disabilities, cultural dimensions); and d) Culturally appropriate processes and protocols previously established in the KMM project. Further, a key focus of the research team is to build capacity as the programme has a number of junior academic and community researchers.

#### Co-design approaches and research

The choice of the two projects occurred during initial co-design meetings. The opportunity to be part of a larger network was expressed by providers as an enabler for co-creating innovative models that address the current challenges faced by kaumātua. Both projects will be developed through an approximately 6 to 9-month planning and co-design process with service providers. Experience from a previous project suggests that this is the right amount of time [37, 94] for appointing personnel, forming an advisory board, and recruiting participants. To enable a robust co-design process, each service provider will be resourced to appoint a community researcher who will support the administration of the project. KMMP researchers will provide initial documents and facilitate the co-design process; each project will include at least two of the five leadership team members, who all have experience with co-design processes. A fidelity check during the co-design process will also be included to assess whether the approach is being delivered and used as designed.

#### Research design

The research design for each project is a mixed-methods, pre-test and two post-test, staggered design with 2–3 providers receiving the approach first and then 2–3 receiving it on a delayed basis. A pre-test (baseline) of all participants will be completed. The approach will then be implemented with the first providers. There will then be a follow-up data collection for all participants (post-test 1). The second providers will then implement the approach, which will be followed by a final data collection for all participants (post-test 2). This BAG-approved research design enables a rigorous comparison of the approaches whilst ensuring that all participants receive the approach, which is important to a kaupapa Māori methodology [85], and is also considered a novel, strong, ethical, and pragmatic design for approaches in the health service sector [95]. Discussions regarding the design will be on-going, particularly in relation to implementation, to ensure cultural appropriateness and research integrity are maintained throughout the research. It was decided not to include random assignment of providers to the projects, in order to respect the service providers’ choice in participation and to demonstrate a commitment to co-design. However, providers will be randomly allocated to the first or second groups. Previous research with Māori communities and providers shows that research projects that privilege a research model compared to a service model have significant challenges in administering the research [96]. Our research approach acknowledges those findings and seeks to offer an alternative “gold standard” research design for working with kaumātua and Māori communities.

#### Data collection measures/procedures

A common set of quantitative and qualitative measures will address the core themes of hauora and mana motuhake, mostly using scales validated from the previous KMM research [36, 97]. For hauora (hinengaro [mental], tinana [physical], wairua [spiritual], and whanaunga [social]), the following items/scales will be used: self-reported health [98, 99], mental/physical health-related quality of life (HRQOL) [100, 101], spiritual wellbeing [102], loneliness [2, 103], perceived and desired social support [104], and cultural connection [40]. Mana motuhake will be measured via perceived autonomy [105], life satisfaction [106], sense of purpose [107], and historical trauma [65]. See Table 1 for a list of constructs and measures. Qualitative data will be formed from open-ended questions about impressions and impacts of the intervention on the same instrument. The total number of items in the common survey excluding demographics will be 29; this allows approximately 15–20 items related to specific projects without overburdening kaumātua. All measures will be revisited during the co-design process to ensure relevance and appropriateness, particularly using the knowledge and experience of the advisory group members and providers. Up to two focus groups with participants will be hosted at each location, with another provider focus group dedicated to provider-level outcomes relating to specific projects. This mix of quantitative and qualitative data provides a robust data set that supports triangulation of the research findings.

**Table 1:** Constructs and Measures Common to both Projects

Construct	Measures	Number of Items
***Pre- and Post-Test Measures***		
Hauora—tinana/hinengaro	Self-rated health	1
Hauora—tinana/hinengaro	Health related quality of life—physical and mental wellbeing	7
Hauora-wairua	Spiritual wellbeing	1
Hauora-whanaungatanga	Loneliness	4
Hauora-whanaungatanga	Cultural connection	3
Hauora-whanaungatanga	Social support	4
Mana motuhake	Perceived autonomy	1
Mana motuhake	Global life satisfaction	1
Mana motuhake	Sense of purpose	3
Mana motuhake	Historical trauma	4
Demographics	Living situation, relational status, gender, age, iwi (tribe)	5

The participants can complete the questionnaire on their own or via a structured interview administered by a Māori research team member and can have a support person present if desired (data will be collected on questionnaires to assess potential bias). The questionnaire will have both te reo Māori and English language versions. The te reo Māori version will involve a translation and back-translation procedure to ensure equivalence to the English version. The questionnaires will be written with plenty of space and large font to support the reading needs of kaumātua. Focus groups will be conducted at the end of the approach and are able to be held in either English or Te Reo Māori. Consistent with previous research, participants will receive a $50 koha for each data collection point and related events.

#### Cultural safety and research ethics

The research team will seek ethics approval through the University of Waikato’s Human Research Ethics Committee for each project separately. Furthermore, the research team will work with the advisory groups to develop a training procedure around data collection (including secure data management procedures) that ensures participant safety. Given the researchers’ previous experience interviewing kaumātua, a culturally appropriate approach for data collection developed in earlier projects [108, 109] may be used including karakia, whakawhanaungatanga, shared kai, kanohi-ki-te-kanohi, koha and whakawhitiwhiti whakaaro (collective debrief).

#### Data analysis

Prior to conducting the primary data analysis, psychometric properties of the scales will be re-affirmed to ensure there are not regional differences. Psychometric properties of specific scales that have not been validated with Māori populations will also be established within the projects. Factorial validity will be assessed with confirmatory factor analysis and reliability established with Cronbach’s alpha. Analysis will be completed with AMOS 26 [110].

Qualitative data will be analysed using thematic analysis [111] following procedures used in previous work with kaumātua. Each analysis will be undertaken by two research team members, with at least one Māori team member [108, 112], whilst it is also likely kaumātua will be included in this phase of analysis. At the very least, themes will be shared with kaumātua and service providers as a validity check to assess if revisions to the analysis are required.

Quantitative data analysis will include several steps. First, statistical assumptions including patterns of missing data will be assessed. Second, descriptive statistics including means, standard deviations, and confidence intervals for continuous data and frequencies for categorical data will be provided. Finally the analysis will involve multilevel analysis of mixed models following procedures to isolate the effect of the intervention across different groups at different times using average treatment effect on the treated [113]. In addition, we will include nesting of repeated measures within individual teina and teina within intervention group. Analysis will be completed with SPSS 26 [114]. Specific models will be identified for the projects.

Finally, estimates of cost effectiveness will be determined using incremental cost effectiveness analysis (ICEA) [115, 116]. ICEA involves estimating the cost per unit improvement in the outcome variable. There are multiple outcome variables in this research programme; however, because it is reasonable to expect that changes in these variables are highly correlated, using 1–2 variables should be sufficient [117] (likely to be HRQOL [hauora] and sense of purpose [mana motuhake] pending advisory group consultation). ICEA involves estimating the ratio of the increase in the outcome variable (as measured by the estimated effect size from the approach trial) to the estimated average cost per participant of the research programme (the incremental cost-effectiveness ratio; ICER). This calculation assumes that the cost of the status quo (no approach) is zero. ICER is a common measure used in the evaluation of the cost-effectiveness of health approaches [118, 119].

Data analysis will be completed for both projects separately; thereafter, results will be compiled into a collective report to compare the analysis by project (including comparisons of impacts on common outcomes), and to disseminate the findings to various stakeholders. This integration will help service providers and other end-users assess whether specific projects meet the needs of kaumātua.

## Specific research projects

### Project 1 – Tuakana-teina peer education

This project builds on the model from a previous project [36], which resulted in significant gains in social support/connection, life satisfaction/mana motuhake, and HRQOL helping kaumātua prepare for life transitions [36, 37, 94]. While the model was trialled in that project, the current version is a significantly enhanced model with two elements that make it innovative. Firstly, service providers noted the need to test the model with other providers. Secondly, service providers believed that the model could be more effectively applied to kaumātua with greatest needs rather than generally for life transitions, particularly in the context of research showing that Māori are more likely to have unmet needs for primary health care than non-Māori [120]. Through the co-design process the research team will work with each service provider to identify the key health and social issues particular to their community.

Once these are defined and a resource kit is created to guide kaumātua, a small number of kaumātua (*n* = 4) will be purposively selected from each service provider and will take part in a Tuakana Orientation Programme. This programme includes four workshops that offer suggestions for communication skills ground in Te Ao Māori and practice around being a peer educator with specific details identified elsewhere [37]. They will then serve as tuakana (peer educator/supporter) for six teina (recipient of peer education/support) for a period of 6-months. Tuakana will talk with teina up to six times to understand their needs and empower them to gain access to needed health and social services. A community research serves as a coordinator for the project and is a resource for helping tuakana and teina link to specific services. The community researcher also provides a resource booklet of available services in the community that tuakana share with teina during the conversations.

**Fig. 2:**
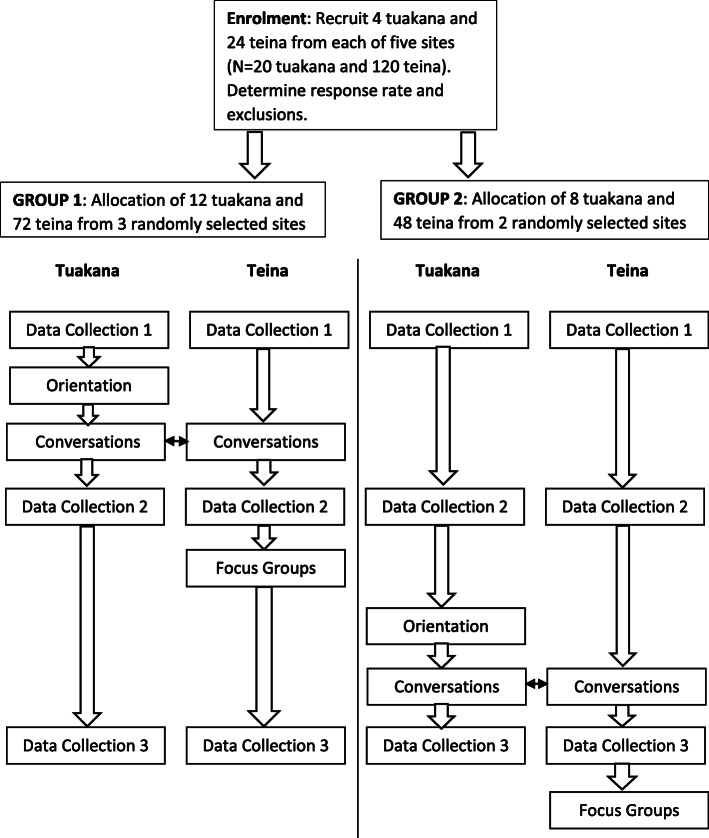
Planned CONSORT Flow Diagram and Data Collection for Tuakana-Teina Project. Note: Participants not allocated to intervention, lost to follow up and excluded from analysis will be calculated at the conclusion of the intervention

Five providers will participate in this project. We will attempt to randomly select teina using a list of registered kaumatua from each provider (*n* = 24 per provider) and exclude participants with advance dementia or cognitive impairment and any other health conditions the provider feel would negative impact participants. Figure 2 provides a flow diagram of proposed recruitment and data collection procedures.

The sample size was determined by a balance of resources and power to determine the optimal design and confirmed with the od.exe software [121]. Twenty tuakana and 120 teina will be recruited in total from the five providers; with all tuakana retained/replaced and 67% of teina retention as occurred in a previous iteration of this model [36] it is expected that at least 20 tuakana and 80 teina will be retained. With the assumption of *p* = .05 and intra-class correlation = .01 (from our previous study) [36], there will be sufficient power (=.80) to identify a medium effect (d = .5) in change over time and a group X time interaction. A medium effect size was chosen given prior research on the impact of elder peer educators on self-rated health and related wellbeing outcomes [122, 123] and our previous research [36].

For specific measures, this project will use a measure of elder abuse adapted from a previous scale [124], housing (two items created for the study; one adapted from the Māori Social Survey) [102], and three items on service use and satisfaction (two created for this study and one from our previous project) [36]. See Additional file 1 for the items created for this study. The project has been prospectively registered with Australia New Zealand Clinical Trial Registry (ACTRN12620000316909); it has also received research ethics approval (HREC2019#81).

To test the objective, a mixed-model regression analysis will be used where the participant responses over the three time periods are nested within individual teina and within providers (i.e., a three-level model). Mean responses for each of the key outcomes will be used as the dependent variables (e.g., HRQOL, life satisfaction, health service utilisation (depression). The model will include the following independent variables: a) within group responses (i.e., responses for three time periods) for change over time; b) intervention received or not to identify intervention effect; and c) random intercepts to account for different teina baseline scores. Co-variates will include any independent variables that differ between the two approach groups at the baseline period. An intent-to-treat analysis will be used for the primary testing. A dose-response model will be considered including number of interactions between tuakana-teina and a rating of conversational quality between the tuakana-teina. Also, it will be examined whether missing data due to loss to follow up can be multiply imputed via chained equations [125].

### Project 2 – Mātauranga Tuku Iho (intergenerational knowledge and language in action)

This project will highlight the role of kaumātua as carriers of mātauranga [21]; specifically, utilising cultural knowledge exchange as a driver for physical activity. The innovation of this project is that it provides an avenue to increased intergenerational exchange and physical activity that is consistent with cultural values and community activities. The pursuit of cultural knowledge for perpetuating/improving subsequent generations is a key theme of cultural narratives [126], such as Tāne’s ascension and descension through the 12 heavens to obtain the baskets of knowledge [127]; and then to share that knowledge for the preservation and perpetuation of whakapapa (genealogy). Māori narratives centre on “ako” where a reciprocal relationship of mutual learning and teaching between kaumātua and whānau (especially mokopuna or grandchildren) leads to “māramatanga” (change and insights) [128].

A key element of mātauranga is te reo Māori and the project will include language lessons for kaumātua who are not fluent speakers. This is especially important in the context of a generation of kaumātua who faced assimilation policies and practices and were discouraged from learning te reo and hence have experienced cultural dissonance. Research shows a link between speaking te reo, and cultural continuity, which have positive health outcomes [51, 129] including a positive association with HRQOL [40]. This approach involves teaching te reo Māori within the context of learning and sharing mātauranga such as mihi, whakapapa (genealogy), karakia (prayer), and waiata (songs).

The expectation is that the project will be divided into a series of wānanga (or group meetings) that provide instructional sessions about language, tikanga and physical activities across a range of skill levels. The sessions will be tailored to the needs of kaumātua given their previous experiences and knowledge about the session content. The content of the wānanga will be developed through a co-design process with providers and kaumātua and will be documented upon completion.

**Fig. 3:**
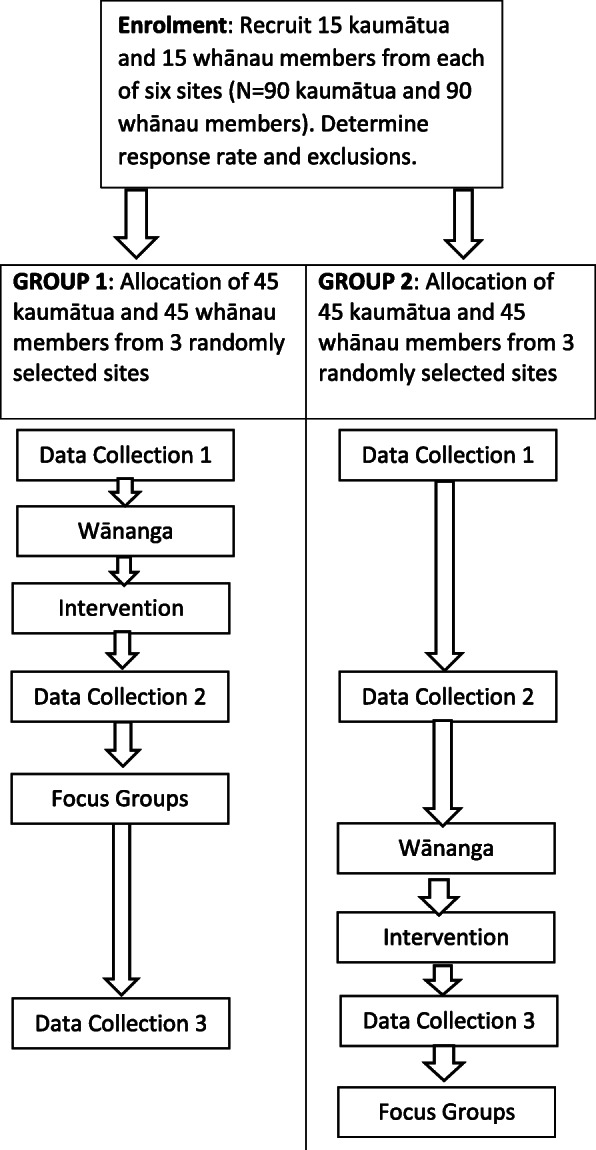
Planned CONSORT Flow Diagram and Data Collection for Project for Mātauranga Tuku Iho. Note: Participants not allocated to intervention, lost to follow up and excluded from analysis will be calculated at the conclusion of the intervention

After the wānanga kaumātua will be asked to engage with at least one member of their whānau to share mātauranga and speak/share reo while participating in a regular activity (e.g., walk to a sacred place; its importance and imperative to preserve the mātauranga of these sacred places is to pass this knowledge on and share stories/pūrākau) that helps improve physical functioning (e.g., participating in Kaumātua Olympics [fun, semi-competitive activities], Iron Kaumātua [triathalon], or simply starting to walk regularly). The intervention is designed to create individually determined improvements on the targeted outcomes. Figure 3 displays a flow diagram of proposed recruitment and data collection procedures.

The specific measures likely will include quality of intergenerational relationships [130], cultural knowledge [102], cultural identity [40], resilience [131], physical functioning [132, 133], and te reo ability. This project will be registered with the Australia New Zealand Clinical Trial Registry, along with completing the application for research ethics after the co-design phase. The project starts 6 months post the start of the tuakana-teina project (originally was 1 year, but COVID-19 restrictions postponed the start of tuakana-teina).

The sample size of 90 kaumātua and 90 whānau members across six providers (*n* = 15 pairs each) was determined by a balance of resources and power to determine the optimal design and confirmed with NCSS-PASS software [134]. Sixty participant dyads are expected to be retained given the 67% retention rate in our previous research [36]. This sample size is sufficient to detect a small to medium effect size (d = .2, power = .80, *p* = .05) in time from pre-test to final post-test and a small to medium effect (d = .3, power = .80, p = .05) for group X time interaction. The chosen effect size is based on a recent study about the impact of small amounts of physical activity on physical functioning for older people [135]. A similar effect is expected for the other specific variables, particularly cultural knowledge for whānau.

To test the objective, a mixed model analysis will be used where the participant responses over time are nested within individuals and nested within providers (i.e., three-level model). Mean responses for each of the key outcomes will be used as the dependent variables (e.g., HRQOL, life satisfaction, physical functioning, cultural knowledge exchange). The model will include the following independent variables: a) within group responses (i.e., responses for three time periods) for change over time; b) intervention received or not to identify intervention effect; and c) random intercepts to account for different teina baseline scores. Co-variates will include any independent variables that differ between the two approach groups at the baseline period. An intent-to-treat analysis will be used for the primary testing. A dose-response model will be considered including the number of interactions between kaumātua and whānau members, the amount of activity engaged in, and measures of the paired relationship of kaumātua and whānau member (e.g., degree of agreement in cultural exchange). It will be determined whether missing data due to loss to follow up can be multiply imputed via chained equations [125].

## Discussion

Epistemologically, kaumātua mana motuhake highlights the strengths and mana (status) of being a kaumātua [136]. It is almost redundant to outline from a customary and philosophical standpoint that Māori culture venerates its elders, such is its centrality to a Māori way of life; from the way that many Māori whānau operate beyond the bounds of a typical nuclear family, to tikanga (cultural practices) observed on marae (community meeting place) and in various community settings. As Sir Hirini Moko Mead states: “[t] he kaumātua and kuia, the elders, are often the guardians of tikanga. They are expected to know” and “[e] xperience is definitely helpful in knowing what to do (p. 14).” [137] Also, as Sir Mason Durie puts it: “[w] hen other New Zealanders might be contemplating withdrawal from public life, older Māori are often encouraged to accept new responsibilities expected by their own people – self-interest will give way to the interests of whānau and hapū (p. 4)” [138].

KMMP takes a strengths-based approach that conceptually reframes the notion of ageing away from a deficit model [139], discourse, which assumes an increasing percentage of older people over the first half of the twenty-first century is coterminous with an increasing burden on the socio-economic system; a discourse underpinned by a deficit model that focuses on isolation, vulnerability, limitations and dependency [136, 140]. The central discourse needs to shift if Aotearoa is to re-comprehend older people as highly valuable members of society. The esteem of elders in Māori culture in principle (if not always in practice) signals transformative potential.

Similarly, one of the issues with the majority of current health research and Indigenous peoples is that it is often validated via a logic of disparity, where Indigenous health statistics are measured against a non-Indigenous baseline. Whilst the logic of disparity helps to define the problem, in doing so it simultaneously defines Indigenous peoples as ‘the problem’ to be fixed and, consequently, falls into the trap of a deficit model framing [141]. In contrast, this research acknowledges that the problem is not kaumātua, rather the comparatively poor Indigenous health statistics are directly related to colonisation and the continued structural discrimination within the health system itself. Although health research in this space is in its infancy, the broader thesis to be tested is that Indigenous cultural revitalisation will increase the wellbeing of Indigenous communities. The cultural dissonance and, therefore, the cultural continuity of Indigenous peoples is an under-investigated concept in health literature, especially in relation to the elderly. This research directly investigates this concept by examining the association between kaumātua culture and health in relation to learning te reo Māori; mātauranga; and tuakana/teina.

The present research, therefore, is original and innovative, and will methodologically contribute to research being carried out in other settler-colonial contexts. Two specific outcomes are anticipated from this research; firstly, it is hoped that the research methodology provides a framework for how government agencies, researchers and relevant sector stakeholders could work with Māori communities. This project will help demonstrate that a participatory approach following the He Pikinga Wairora framework [88] working with end-users, using systems thinking and centring on kaupapa Māori provides a strong approach leading to positive health outcomes and health equity. Secondly, the two individual projects will each produce a tangible approach that, it is anticipated, will be cost effective in enhancing kaumātua hauora and mana motuhake. These products will be made accessible to various end-users and, if effective, will enable advocacy with stakeholders and policy-makers to include them as part of the means to serve the rapidly growing population of kaumātua. The point of the research is, therefore, not to simply accept that the tikanga surrounding Māori elders trickles down to all Māori and indeed to the ageing population in Aotearoa as a whole, but rather to employ cultural concepts to help shift the public discourse surrounding ageing and to contribute to the creation of kaumātua health knowledge so that, firstly, the ageing population is seen to be valued and, secondly, the ageing population is given mana motuhake—the responsibility of taking on new and vital roles as their life situations evolve. As a result, the research objectives are informed by the position that transformative outcomes for kaumātua are symbiotically related to transformative outcomes for the broader Māori communities. These transformative outcomes have the potential to make lasting impacts for Māori communities. The ultimate goal is transformational change in the way kaumātua are recognised, supported, and served by the health system.

## Supplementary information

**Additional file 1.** Items created for this study.

## Data Availability

The datasets used and/or analysed during the current study are available from the corresponding author on reasonable request.

## Glossary

HRQOL: Health-related quality of life
ICEA: Incremental cost effectiveness analysis
ICER: Incremental cost-effectiveness ratio
SRMR: Standardized root mean square residual
Aotearoa: New Zealand
Hauora: Health
Hapū: Subtribe
Hinengaro: Mind, thought, intellect
Iwi: Tribe
Kaupapa Māori: Research by Māori for Māori
Koha: Offering
Mana: Status and prestige
Mana motuhake: Autonomy, identity and self-actualisation
Māori: Indigenous people of New Zealand
Mātauranga: Indigenous knowledge
Pono: Truth, integrity
Te Ao Māori: Māori epistemology
Te Kupenga: Māori Social Survey
Teina: Peer education recipient
Tikanga: Customs and protocols
Tinana: Body
Tino rangatiratanga: Independence and autonomy
Tuakana: Peer educator
Tuakana-teina: Older sibling/younger sibling
Wairua: Spirit
Whakawhanaungatanga: Making social connections
Whānau: Extended family
Whanaungatanga: Social health
