# Supplementary material for: Kaumātua Mana Motuhake Pōi: a study protocol for enhancing wellbeing, social connectedness and cultural identity for Māori elders
Source: BMC Geriatr. 2020 Oct 2;20:377. doi: 10.1186/s12877-020-01740-3 (PMC7530863; doi:10.1186/s12877-020-01740-3)
Supplement: Supplementary file 1 — Additional file 1. Items created for this study. [file 12877_2020_1740_MOESM1_ESM.docx]

**Items Written for the Study**

*Housing*

1. How much of a problem is the financial aspects of your housing situation such as affording to live in a stable housing situation (not a problem, small problem, a medium-sized problem, a bit problem)?
2. How much of a problem is your housing situation to have autonomy, such as have room or space of your own (not a problem, small problem, a medium-sized problem, a bit problem)?

*Services*

1. How much knowledge would you say you have about services that are available to you? (very much, some, a little, not at all)

1. How much do health and social services help you with your needs? (very much, some, a little, not at all)
